# Complementation of diverse HIV-1 Env defects through cooperative subunit interactions: a general property of the functional trimer

**DOI:** 10.1186/1742-4690-6-75

**Published:** 2009-08-11

**Authors:** Karl Salzwedel, Edward A Berger

**Affiliations:** 1 Laboratory of Viral Diseases, National Institute of Allergy and Infectious Diseases, National Institutes of Health, Bethesda, MD 20892, USA; 2 Current address: Division of AIDS, NIAID, 6700-B Rockledge Drive, Room 4149, Bethesda, MD 20892, USA

## Abstract

**Background:**

The HIV-1 Env glycoprotein mediates virus entry by catalyzing direct fusion between the virion membrane and the target cell plasma membrane. Env is composed of two subunits: gp120, which binds to CD4 and the coreceptor, and gp41, which is triggered upon coreceptor binding to promote the membrane fusion reaction. Env on the surface of infected cells is a trimer consisting of three gp120/gp41 homo-dimeric protomers. An emerging question concerns cooperative interactions between the protomers in the trimer, and possible implications for Env function.

**Results:**

We extended studies on cooperative subunit interactions within the HIV-1 Env trimer, using analysis of functional complementation between coexpressed inactive variants harboring different functional deficiencies. In assays of Env-mediated cell fusion, complementation was observed between variants with a wide range of defects in both the gp120 and gp41 subunits. The former included gp120 subunits mutated in the CD4 binding site or incapable of coreceptor interaction due either to mismatched specificity or V3 loop mutation. Defective gp41 variants included point mutations at different residues within the fusion peptide or heptad repeat regions, as well as constructs with modifications or deletions of the membrane proximal tryptophan-rich region or the transmembrane domain. Complementation required the defective variants to be coexpressed in the same cell. The observed complementation activities were highly dependent on the assay system. The most robust activities were obtained with a vaccinia virus-based expression and reporter gene activation assay for cell fusion. In an alternative system involving Env expression from integrated provirus, complementation was detected in cell fusion assays, but not in virus particle entry assays.

**Conclusion:**

Our results indicate that Env function does not require every subunit in the trimer to be competent for all essential activities. Through cross-talk between subunits, the functional determinants on one defective protomer can cooperatively interact to trigger the functional determinants on an adjacent protomer(s) harboring a different defect, leading to fusion. Cooperative subunit interaction is a general feature of the Env trimer, based on complementation activities observed for a highly diverse range of functional defects.

## Background

The envelope glycoprotein (Env) of human immunodeficiency virus type 1 (HIV-1) promotes virus entry by catalyzing direct fusion between the virion membrane and the target cell plasma membrane; similarly, Env-expressing cells can fuse with target cells to form multinucleated giant cells (syncytia). Env is synthesized as a gp160 precursor protein that assembles into homo-trimeric complexes in the endoplasmic reticulum. During transport through the secretory pathway, gp160 is cleaved in the trans-Golgi network by a furin-like protease(s) to yield the external gp120 subunit noncovalently associated with the gp41 transmembrane subunit (derived from the N- and C-regions of gp160, respectively) [1]. The functional Env spike on mature virions of HIV-1 and the related simian immunodeficiency virus consists of a homo-trimer of gp120/gp41 hetero-dimers [2].

Env-mediated fusion involves a strict division of labor between the two subunits: gp120 is responsible for sequential binding to specific target cell receptors, first to CD4 and then to the coreceptor (a specific chemokine receptor, typically CCR5 or CXCR4); receptor binding then triggers gp41 to promote membrane fusion. These steps involve a tightly orchestrated series of conformational changes in both Env subunits that drive the fusion process. The emerging understanding of the complexities of HIV Env/receptor interactions and the subsequent events leading to fusion/entry have been the central focus of numerous review articles over the past decade [3-8]. X-ray crystallographic analyses of gp120 from HIV-1 [9] and the closely related simian immunodeficiency virus [10] have revealed that CD4 binding induces a profound rearrangement of the relatively disordered gp120 subunit to create a new surface consisting of four anti-parallel beta strands derived from discontinuous regions of the linear sequence; this highly conserved "bridging sheet", which is not present in the unliganded pre-CD4-bound state, is directly involved in binding to coreceptor [11] in conjunction with the third variable loop (V3) of gp120, which determines coreceptor specificity [12,13]. Binding of gp120 to coreceptor then triggers the fusogenic activity of gp41 in a process believed to involve insertion of the gp41 N-terminal fusion peptide (FP) into the target cell plasma membrane [14,15]. Detailed structural information is not yet available for the native state of gp41, but the structure of the final post-fusion state has been determined to be a trimer of hairpins in the form of a six-helix coiled-coil bundle [16-18]. A transient intermediate conformation is thought to exist in which the gp41 subunits adopt an extended triple-helix coiled-coil with the N-terminal FPs inserted into the target cell membrane. The heptad repeat (HR) segments near the external C-terminal region (HR2) then fold to insert in anti-parallel fashion into the grooves formed by the cluster of the three N-terminal heptad repeat (HR1) segments; the resulting formation of a 6-helix bundle brings the virion and target cell plasma membranes together, and provides the driving force for membrane fusion underlying HIV entry. The molecular complexity of the HIV entry process presents a variety of targets for novel antiviral agents [19-22]; the T-20 peptide (enfuvirtide, Fuzeon) targeting the gp41 intermediate conformation is the first-in-class HIV-1 fusion inhibitor [23], and the recently approved maraviroc is the first-in-class inhibitor that binds to the CCR5 coreceptor and blocks the gp120 interaction [24].

While each gp120/gp41 hetero-dimeric complex contains all the determinants required for fusion, it is possible that molecular interactions between complexes within the trimer influence Env function. In a previous study we used a quantitative vaccinia expression-based cell fusion assay to demonstrate that individual subunits within the Env trimer can interact cooperatively during fusion [25]. By coexpressing Env proteins with defects in different essential determinants, we found that functional complementation could occur between subunits within a mixed trimer. In the present report, we show that subunit complementation is a general capacity of the HIV-1 Env trimer, though its efficiency and detectability are dependent on the particular defective variants examined and the assay systems employed. The results are discussed in terms of potential biological implications for Env function and HIV neutralization.

## Methods

### Construction and expression of Env variants

For vaccinia virus expression-based cell fusion assays, HIV-1 Envs were transiently expressed from pSC59-based plasmids under control of a strong synthetic vaccinia virus early/late promoter [26]. Previously described plasmids [27,28] were used to express wild-type Envs from the following HIV-1 strains: LAI [29] (LAV isolate, unless indicated otherwise), plasmid pCB-41; SF162, plasmid pCB-32; Ba-L, plasmid pCB-43, and CM235, plasmid pCB-52. In addition, a Kpn I-Xho I fragment encoding wild-type YU-2 Env was substituted into a variant of pCB-41 containing a unique Xho I site at the 3' end of Env (pKS-9) to create the plasmid pKS-10. As a negative control, an uncleaveable (Unc) mutant form of LAI (IIIB isolate) was used (plasmid pCB-16).

**Figure 1:**
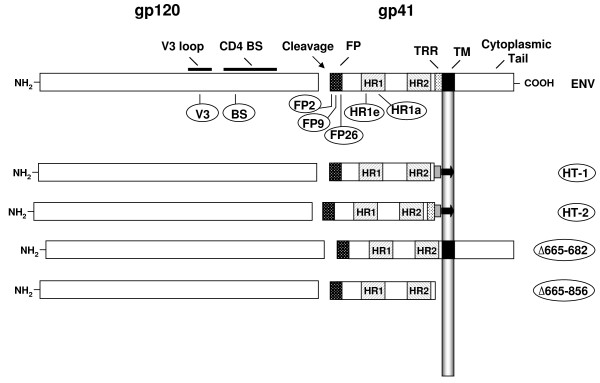
**Mutations in HIV-1 Env**. A schematic representation of HIV-1 Env. Functional and structural domains within the gp120 and gp41 subunits are labeled at the top: V3 loop (3^rd ^variable loop), CD4 BS (CD4 binding site), FP (fusion peptide), TRR (tryptophan-rich region), TM (transmembrane domain). For various inactivating mutants in the LAI Env (designations encircled), the approximate locations of specific point mutants are indicated underneath, and the deletion mutants are indicated on the right. The specific point mutations are: V3 (R320G in the conserved GPGR motif at the tip of the V3 loop); BS (D368R within the CD4 binding site); various positions in the fusion peptide including FP2 (Val→Glu), FP9 (Leu→Arg) and FP26 (Leu→Arg); heptad repeat mutations HR1e (V570R) and HR1a (I573P). HT-1 and HT-2 are chimeric LAI Env/Thy-1.1 glycoproteins that are membrane-associated via a glycosyl-phosphatidylinositol (GPI) anchor. HT-1 contains the gp41 ectodomain minus the tryptophan-rich region (K665 through I682), whereas HT-2 contains the entire gp41 ectodomain; both constructs have 22 intervening amino acid residues from the C-terminus of Thy1.1. The Δ665-682 construct has a selective deletion of the TRR (K665 through I682) and the Δ665-856 construct has an introduced premature stop codon that results in deletion of the C-terminal 192 aa of Env, including the TM and cytoplasmic domains. See Methods for construction and references.

Plasmids pKS-3 and pKS-4 [25] encode mutants of LAI Env (HXB2 isolate) with a D368R substitution in the CD4 binding site (BS) of gp120 that abolishes CD4 binding (LAI-BS) and a Leu to Arg substitution at residue 26 of the gp41 N-terminal fusion peptide (LAI-FP26) [30], respectively. Additional site-directed mutations were introduced (QuikChange kit, Stratagene, La Jolla, CA) into pCB-41 encoding wild-type LAI Env resulting in the following plasmid constructs. See Fig. 1 Legend for descriptions: FP mutants LAI-FP2 (plasmid pKS-13) and LAI-FP9 (plasmid pKS-14); heptad repeat mutants LAI-HR1a (plasmid pKS-15) and LAI-HR1e (plasmid pKS-16); V3 loop mutant LAI-V3 (plasmid pKS-17). A Kpn I-Bam HI fragment encoding the LAI-Δ665-856 mutant, previously referred to as Δ192 [31], was substituted into a variant of pCB-41 (pKS-8) in which a Bam HI site upstream of the promoter had been destroyed by cutting and then filling in with T4 DNA polymerase; the resulting plasmid was pKS-18. Kpn I-Xho I fragments encoding the mutants LAI-HT-1 [32], LAI-HT-2 [32], and LAI-Δ665-682 [33,34] were substituted into the plasmid pKS-8 to create the plasmids pKS-19, pKS-20, pKS-21, and pKS-22, respectively. Previous studies indicate that each of these variants is capable of Env processing (except for Unc), surface expression (except for LAI-Δ665-856, which is secreted), and CD4 binding (except for LAI-BS) [25,30,32-34].

For MAGI cell HIV infectivity assays, virus was expressed from the pNL4-3 proviral clone [35] encoding wild-type LAI Env (LAV isolate). pNL4-3 containing a frame-shift mutation at the Nhe I site within Env (pNL4-3Δenv) [33] was used as a negative control. Nhe I-Bam HI fragments encoding the LAI-FP26 and LAI-BS mutants [25] were subcloned into pNL4-3 to create pKS-11 (encoding NL4.3-FP26) and pKS-12 (encoding NL4.3-BS), respectively. The phenotype for each construct, both when expressed alone and in complementation experiments, was confirmed using two independent plasmid clones constructed in parallel.

### Vaccinia virus-based cell fusion assay

Env-mediated cell fusion activity was measured using a quantitative vaccinia-based reporter gene assay as described previously [36,37]. Each vaccinia virus was used at a multiplicity of infection of 10. Target cells were prepared by co-infecting NIH 3T3 cells with vaccinia virus recombinant vCB21R-LacZ containing the *E. coli *lacZ reporter gene linked to the T7 promotor [38], plus vaccinia recombinants encoding the following cDNAs linked to vaccinia early/late promoters: CD4, vCB-3 [39] and the designated coreceptor CCR5, vHC-1 [40] or CXCR4, vCBYF1-fusin [41]. Effector cells were prepared by transfecting HeLa cells with the above-described plasmids containing the Env genes linked to a strong synthetic vaccinia early/late promoter and infecting with vaccinia recombinant vP11T7gene1 encoding bacteriophage T7 RNA polymerase [42]. Transfection was performed with DOTAP (Boehringer Mannheim, Indianapolis, IN); the total amount of DNA was held constant at 5 μg DNA per 25 cm^2 ^flask, in both single-transfection and cotransfection experiments. Effector and target cells were incubated overnight at 31°C to allow expression of the recombinant proteins. After these cells were washed by centrifugation, they were mixed in equal numbers in duplicate wells of a 96-well plate (2 × 10^5 ^of each per well) and incubated for 2.5 hr at 37°C. Fusion reactions were terminated by addition of nonidet-P40 (0.5% final) and quantified by spectrophotometric measurement of β-galactosidase activity as described previously [36]. For each data point, error bars indicate the standard errors of the mean of duplicate samples; in cases where error bars appear to be absent, the data points were so close that error bars are not visible. All experiments were repeated at least twice; representative data are shown for each experiment.

### MAGI cell assays for cell fusion and virus entry

HIV-1 entry and Env-mediated cell fusion in the context of HIV-1 provirus expression were measured using the HeLa-CD4/LTR-β-gal (MAGI) indicator target cell line [43], which was obtained from the NIH AIDS Research and Reference Reagent Program (originally contributed by M. Emerman). BS-C-1 cells plated at 3 × 10^5 ^per well in 6-well plates the previous day were transfected (or cotransfected) with the designated pNL4-3-based proviral construct(s) using FuGENE 6 (Boehringer Mannheim, Indianapolis, IN) according to the manufacturer's protocol. The next day, cells were washed and given fresh media (2 ml per well) containing 10 mM HEPES. Three days post-transfection, the supernatants were removed, filtered through a 0.45 μm filter to remove cellular debris, and stored at 4°C. For cell fusion assays, the cells were trypsinized, washed, mixed 1:10 with MAGI cells, and replated in duplicate at 1 × 10^5 ^total cells per well of a 24-well plate. Cells were allowed to fuse overnight at 37°C and were then stained with X-gal. Cell fusion was quantitated by counting the total number of blue multi-nucleated syncytia per well with the aid of a grid. For the virus entry assays, p24 levels in the filtered supernatants were quantitated using the HIV-1 p24 Antigen Assay (Coulter), and supernatant volumes were normalized accordingly. MAGI cells were infected in duplicate with 300 μl of filtered supernatant per well of a 24-well plate and stained with X-gal 48 hrs post-infection. Virus entry was quantitated by counting the total number of blue foci per well. For complementation pairs, supernatants containing up to 2.35 ng of p24 per well were used (equivalent to 628 infectious units for wild-type). This corresponds to approximately 15–20% of the total supernatant from the cells. For each data point, the standard errors of the mean of duplicate samples are shown.

## Results

To test the ability of fusion-inactive Env subunits to functionally complement one another in the context of mixed Env trimers, we first employed a vaccinia-based quantitative cell fusion assay system wherein fusion between effector cells expressing Env and target cells expressing the necessary receptors leads to reporter gene activation (β-galactosidase production) [36,37]. We examined complementation between variants in gp120 that were inactive due to inability to interact with CD4 (CD4 BS mutation) or coreceptor (mismatched specificity, or mutation in the V3 loop), as well as variants in gp41 with mutations at different points within the FP and HR1 regions, as well as modifications of the membrane proximal tryptophan-rich region (TRR) and the transmembrane (TM) domain (Fig. 1). Throughout these studies, target cells lacking coreceptor served as negative controls; where indicated, an uncleaveable mutant Env (Unc) containing a mutation in the gp120/gp41 cleavage site provided an additional negative control.

### Complementation by Env subunits from HIV-1 primary isolates of different genetic subtypes

**Figure 2:**
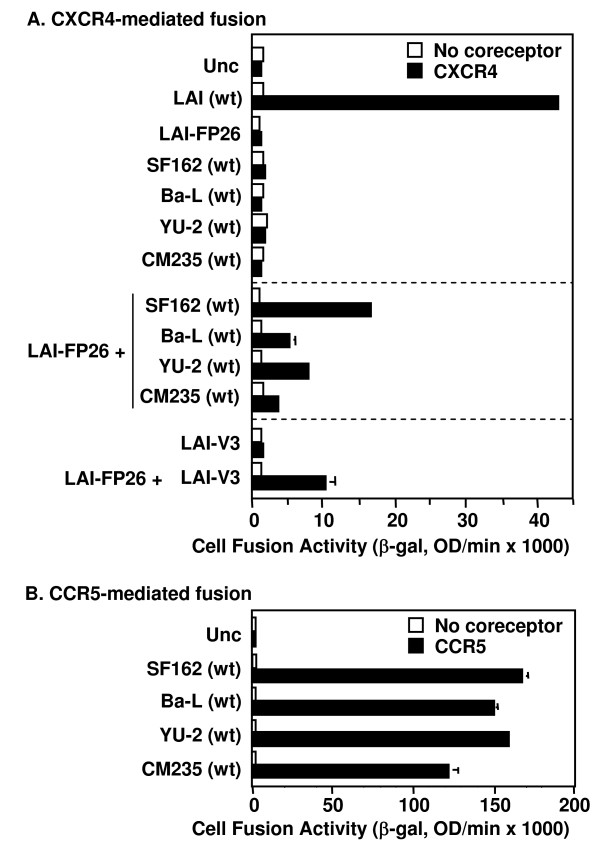
**Complementation with laboratory-adapted and primary Envs from different clades**. The vaccinia system was employed to assay cell fusion between effector cells expressing Envs and target cells expressing CD4 either with (filled bars) or without (open bars) the indicated coreceptor. **A) **CXCR4-dependent fusion. Effector cells expressed the indicated Envs either individually (top section) or in combination with LAI-FP26 (middle section). Effector cells expressed LAI-V3 individually or in combination with LAI-FP26 (bottom section). **B) **CCR5-dependent fusion. The indicated wt Envs were assayed for their intrinsic fusogenicity with target cells expressing CD4 and CCR5.

Previously we demonstrated complementation between Env constructs from two HIV-1 strains that were highly laboratory-adapted and both clade B: LAI (X4, i.e. CXCR4-specific) and SF162 (R5, i.e. CCR5-specific) [25]. To determine whether complementation potential is a more general property of HIV-1 Envs, we analyzed the relative complementation efficiencies of an LAI Env mutant containing a defective FP (LAI-FP26) with Envs from diverse R5 isolates in a CXCR4-dependent cell fusion assay. When tested alone under these conditions, wild type LAI showed potent activity whereas the LAI-FP26 mutant and all four wild type R5 Envs were non-fusogenic (Fig. 2A, top section). In coexpression experiments that enabled mixed trimer formation, complementation of LAI-FP26 with the R5 Envs occurred not only with SF162 as shown previously, but also with the laboratory-adapted Ba-L strain (clade B) and the primary YU-2 (clade B) and CM235 (CRF01_AE recombinant) isolates (Fig. 2A, middle section). The differences in the relative complementation efficiencies of the various Envs correlated roughly with their relative intrinsic fusogenicities in a CCR5-dependent assay (Fig. 2B).

### Complementation by Env subunits containing a mutationally inactivated V3 loop

Our previous results [25] coupled with the data above demonstrate that an Env with a mutational defect in gp41 can complement an Env containing a gp120 subunit incapable of interacting with coreceptor due to mismatched coreceptor specificity. We wished to extend this finding by testing a gp120 subunit rendered inherently defective for coreceptor interaction by site-directed mutation. The V3 loop, though highly variable, contains a conserved β-turn motif at its crown (typically GPGR or GPGQ) that is essential for coreceptor binding activity [12,13]. We analyzed a point mutant (LAI-V3) containing a G in place of the R residue in the GPGR motif, which has been shown previously to abolish fusogenicity [44]. Our results demonstrate that the fusion-defective LAI-V3 was able to complement LAI-FP26 (Fig. 2A, bottom section). The fusion activity was in the same range observed for the coreceptor-mismatched Envs (Fig. 2A, middle section), indicating that complementation efficiency was not limited by structural incompatibilities between Envs from these different strains.

**Figure 3:**
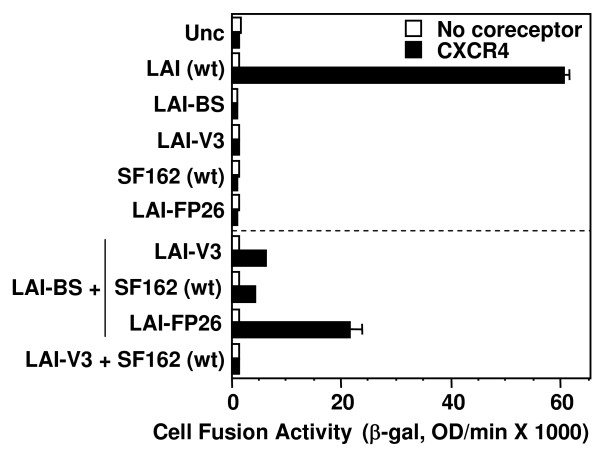
**Complementation between gp120 variants**. The vaccinia system was employed to assay cell fusion between effector cells expressing Envs and target cells expressing CD4 either with (filled bars) or without (open bars) CXCR4. Effector cells expressed the indicated Envs either individually (top section) or in combination with the indicated gp120-defective Envs LAI-BS or LAI-V3 (bottom section).

Previously we demonstrated complementation between Envs containing different nonfunctional gp120 subunits within a mixed trimer; functional mixed trimers were formed when LAI-BS (defective for CD4 binding) was coexpressed with wild-type SF162 (incapable of coreceptor interaction in a CXCR4-specific assay) [25]. To extend this finding we tested the ability of LAI-BS to complement LAI-V3, i.e. gp120 subunits incapable of interacting with CD4 and coreceptor, respectively (Fig. 3). The efficiency was similar to that observed for complementation between LAI-BS and SF162, again indicating that there were minimal structural incompatibilities in mixed trimers between these two strains. As reported previously [25], these examples of complementation between Envs with distinct gp120 receptor binding deficiencies were somewhat less active than complementation between Envs containing a defective gp120 and a defective gp41 (LAI-BS + LAI-FP26) (Fig. 3). As expected, no complementation was observed upon coexpression of LAI-V3 with SF162, since the gp120 from neither Env is capable of functioning with the CXCR4 coreceptor.

### Varying complementation efficiencies of different point mutations within the gp41 FP

**Figure 4:**
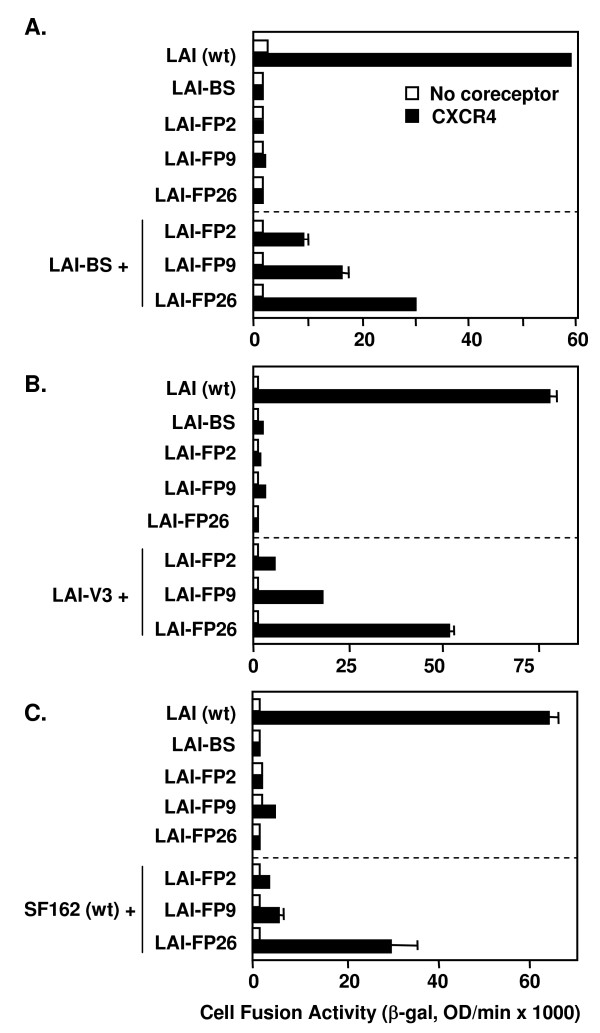
**Complementation with Envs containing point mutations in the gp41 fusion peptide**. The vaccinia system was employed to assay cell fusion between effector cells expressing Envs and target cells expressing CD4 either with (filled bars) or without (open bars) CXCR4. Within each section, effector cells expressed the indicated Envs either individually (top section) or in combination with the indicated nonfunctional Envs (bottom section): A. LAV-BS, B. LAV-B3, C. SF162 (wt).

Our previously described data and the experiments above demonstrated functional complementation of a particular gp41 FP mutation, i.e. substitution of Arg for Leu at the 26^th ^position from the gp41 N-terminus (LAI-FP26). To extend these analyses, we analyzed two additional FP mutations previously shown by others to abolish fusogenic activity without affecting Env processing or CD4 binding [30]: LAI-FP2 substitutes Glu for Val at the 2^nd ^position of the FP, and LAI-FP9 substitutes Arg for Leu at the 9th position (Fig. 1). The LAI-FP2 mutant has been shown to dominantly interfere with cell fusion when coexpressed with wild-type Env, whereas the LAI-FP9 mutant reduced fusion two-fold and the LAI-FP26 mutant had no negative effect when coexpressed with wild-type Env [45]. The results of complementation experiments with these gp41 FP mutations are shown in Fig. 4. Consistent with previous reports, each mutation alone strongly impaired cell fusion activity compared to wild type (top sections in Figs. 4A–C). The relative efficiencies of complementation, FP26 > FP9 > FP2 was observed whether the complementation partner was LAI-BS (Fig. 4A), LAI-V3 (Fig. 4B), or SF162 (wt) (Fig. 4C).

### Complementation with gp41 subunits lacking the normal membrane anchoring and membrane proximal external regions

Highly conserved regions close to the membrane are known to be critical for Env function, including the 22 amino acid TM domain that anchors Env to the surface of virions and infected cells, and the membrane-proximal external region, generally defined as the last 24 C-terminal residues of the gp41 ectodomain (L_660 _– K_683_) [15]. This region contains the TRR (defined here as K_665 _– K_683_) and contains or overlaps the epitopes for the broadly neutralizing 2F5 and 4E10 monoclonal antibodies. We tested five previously described defective mutants in this gp41 region for their ability to support complementation. HT-1 and HT-2 are chimeric LAI Env/Thy-1.1 glycoproteins that are membrane-associated via a glycosyl-phosphatidylinositol (GPI) anchor. HT-1 contains the gp41 ectodomain minus the tryptophan-rich region whereas HT-2 contains the entire gp41 ectodomain; both constructs have 22 intervening amino acid residues derived from the C-terminus of Thy1.1 [32]. HG-1 is analogous to HT-1 except that a minimal GPI attachment signal has been used without the intervening Thy-1.1 residues (K. Salzwedel and E. Hunter, unpublished data). LAI-Δ665-856 contains a stop codon in place of the Lys at position 665, resulting in deletion of 192 amino acids from the C-terminus, including the entire tryptophan-rich, TM, and cytoplasmic domains; this protein is secreted into the medium [31]. Finally, LAI-Δ665-682 contains an 18-amino acid deletion of the tryptophan-rich region (K_665 _– I_682_) [33,34].

**Figure 5:**
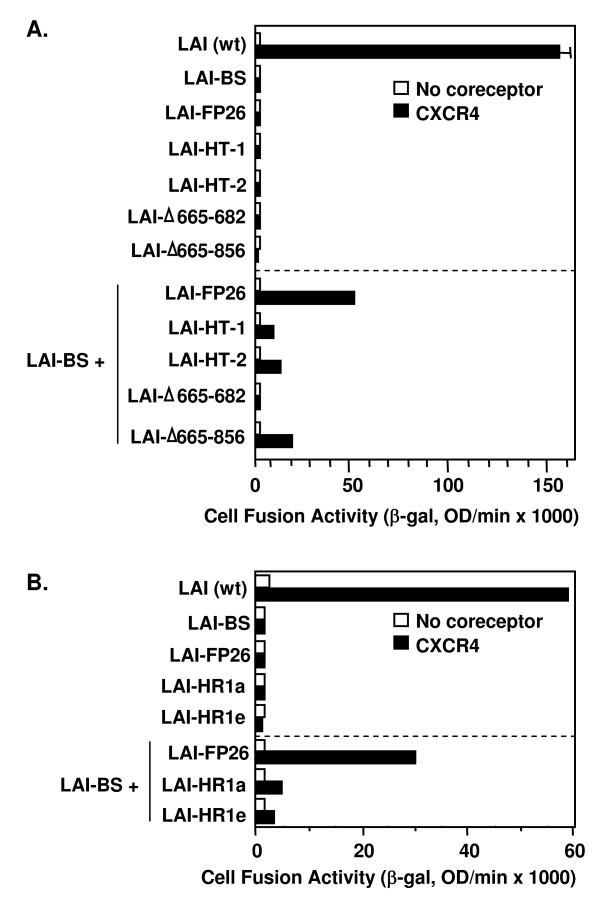
**Complementation with Envs containing alterations in the membrane-spanning domain, the TRR, and HR1**. The vaccinia system was employed to assay cell fusion between effector cells expressing Envs and target cells expressing CD4 either with (filled bars) or without (open bars) CXCR4. Within each section, effector cells expressed the indicated Envs either individually (top section) or in combination with the nonfunctional LAV-BS (lower section).

The results of complementation experiments with these mutants are shown in Fig. 5A. Each of the fusion-defective GPI-anchored Envs was capable of complementing LAI-BS. Perhaps surprisingly, even the truncated non-anchored LAI-Δ665-856 Env was able to complement the full-length LAI-BS665-856 mutant, with comparable or higher efficiency compared to the GPI-anchored constructs.

### Complementation with gp41 subunits containing heptad repeat mutations

Two previously characterized point mutations within the N-terminal heptad repeat region (HR1) of the gp41 ectodomain (Fig. 1) were analyzed for complementation, i.e. substitution of Pro for Ile at residue 573 at the "a" position within the HR1 heptad repeat motif (LAI-HR1a) and Arg for Val at residue 570 at the "e" position within the HR1 heptad repeat (LAI-HR1e). The LAI-HR1a mutation has been shown previously to disrupt self-association of HR1 to form the trimeric coiled-coil pre-hairpin intermediate structure [46] and the LAI-HR1e mutation is suspected to block association of HR2 with the HR1 trimer to form the 6-helix coiled-coil hairpin structure [47]. Interestingly, each of these mutants displayed some complementation activity with LAI-BS Fig. 5B). However the efficiency was relatively low, and no significant complementation by these mutants was observed with Envs defective in CXCR4 interaction (LAI-V3 and SF162 wt, data not shown).

### Complementation requires coexpression of Env mutants within the same cell

**Figure 6:**
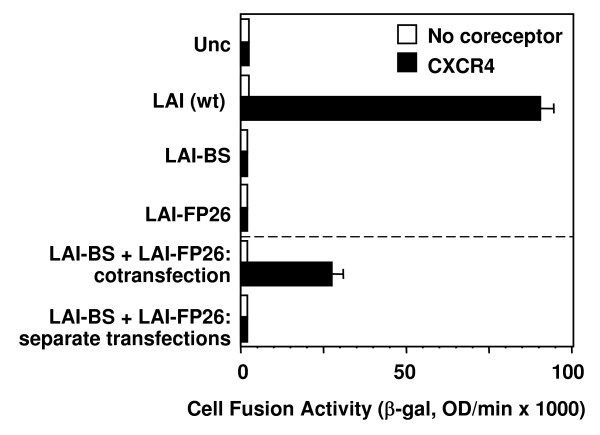
**Complementation requires coexpression of Env mutants within the same cell**. The vaccinia system was employed to assay cell fusion between effector cells expressing Envs and target cells expressing CD4 either with (filled bars) or without (open bars) CXCR4. Top section: Effecter cells expressing the indicated Envs individually. Bottom section: Effector cells either cotransfected with the indicated Envs, or infected separated and mixed 1:1.

The observed complementation activities involved coexpression of two distinct nonfusogenic Env variants within the same cell. Although in our previous study [25] we verified the formation of mixed trimers between the two variants, we could not rule out the possibility that the complementation activity was due to cooperative interactions between nonfusogenic homo-trimers of each variant. While this seemed an unlikely explanation, it has been reported that cell fusion can occur when CD4 and coreceptor are expressed on separate target cells [48]. To determine whether this might also be true for Env trimers expressed on separate cells, we expressed LAI-BS and LAI-FP26 in separate effector cells and asked whether mixing the two cell populations with target cells could result in fusion. As shown in Fig. 6, fusion was detected only when the constructs were cotransfected into the same effector cell population, indicating that functional complementation requires both Env variants to be expressed within the same cell.

### Complementation activity is dependent on the nature of the functional assay

The cell fusion assay used in the above experiments employed vaccinia virus expression technology to produce Env and CD4 and to provide the reporter gene activation system for readout. We wished to assess whether complementation could also be detected in a more biologically relevant situation, i.e. under conditions of HIV-1 proviral expression, using a target cell reporter system more commonly used to quantitate this process. Furthermore, we asked whether complementation can be detected not only by measuring Env-mediated cell fusion, but also virion entry. To address these questions, we expressed Env from molecular variants of an HIV-1 infectious molecular clone and analyzed both cell fusion and virus entry using as targets the well studied HeLa-CD4/LTR-β-gal (MAGI) indicator cell line [43]. Proviruses encoding the LAI-FP26 and the LAI-BS mutant Envs were cotransfected into BS-C-1 producer cells. This Env variant pair was selected because it consistently yielded the highest levels of fusion complementation in the vaccinia-based system. Two alternative assays were then compared. First, the BSC-1 producer cells were used as effectors and mixed with MAGI target cells in a cell fusion assay; second, filtered supernatants from the BSC-1 producer cells containing cell-free HIV-1 virions were used to infect MAGI cells in a parallel virus entry assay. In both cases, complementation was assessed by counting the number of blue foci observed upon *in situ *staining with X-gal. As shown in Table 1, functional complementation was detected in the cell fusion assay: cells transfected individually with either NL4-3-FP26 or NL4-3-BS infectious molecular clones were fusion-incompetent, whereas cells cotransfected with both gave significant fusion activity. By contrast, no complementation was observed in the virus entry assay using the viruses produced from these same cells (Table 1).

**Table 1:** Complementation analysis of Envs expressed from infectious HIV-1 molecular clones in assays of cell fusion and virus entry.

	**No. of blue foci per well**	
**Construct**	**Cell fusion**	**Virus Entry**

Mock transfected	0	0

NL4-3 ΔEnv	0	0

NL4-3 (wt)	620 +/- 45	314 +/- 4

NL4-3-FP26	0	0

NL4-3-BS	0	0

NL4-3-FP26 + NL4-3-BS	40 +/- 2	0

## Discussion

The present results extend our earlier findings [25] by demonstrating that the capacity for functional subunit complementation is a general feature of the HIV-1 Env trimer. We interpret our results to reflect cooperative subunit interactions within mixed heterotrimers, consistent with our previous verification that mixed heterotrimers do indeed form upon coexpression of different HIV-1 Env variants, as well as our previous reference to other examples of mixed trimer formation with glycoproteins from different enveloped viruses [25]; however we cannot formally exclude the possibility that the observed complementation activities reflect complex interactions amongst homo-trimers with different defects expressed on the same membrane. In the present work, complementation was observed upon coexpression of Envs from primary as well as laboratory-adapted HIV-1 strains of different genotypes, and with a wide diversity of defects within both gp120 and gp41 (Figs. 2, 3, 4, 5). Thus fusion does not require every gp120 subunit in the trimer to be competent for CD4 or coreceptor binding, nor every gp41 subunit to contain a functional fusion peptide, a normal membrane anchoring region, a native TRR, or a functional HR1 region. We also demonstrate that complementation requires coexpression of the Env variants in the same cell (Fig. 6), and provide further evidence (by virtue of complementation between LAI-BS and LAI-V3, Fig. 3) against the interpretation that the observed complementation requires reassortment of gp120 and gp41 subunits to form homo-trimers composed of completely functional gp120/gp41 protomers.

We interpret complementation as a reflection of cooperative cross-talk between defective protomers within a mixed trimer, whereby the wild type determinants on one protomer transmit structural changes to activate wild type determinants on an adjacent protomer(s), thereby overcoming defects that are otherwise inactivating in the context of homo-trimers. For example when a CD4 BS mutant is coexpressed with coreceptor inactive variant, we propose that CD4 binding to the subunit(s) with functional BS promotes the conformational changes required for coreceptor binding, which are then transmitted to an adjacent gp120 subunit(s) that can then undergo the essential coreceptor interaction, thereby triggering activation of the wild type gp41 subunits. Particularly striking is the wide range of gp41 mutants capable of complementation when coexpressed with a nonfunctional gp120 variant. Nearly all tested displayed some level of activity, the only exception being LAI-Δ665-682, a normally anchored form containing a deletion of the TRR. The mere absence of the TRR cannot be the simple explanation, since several constructs lacking this region did show complementation activity (HT-1, LAI-Δ665-856). Perhaps misalignment of the LAI-Δ665-682 mutant with wild type gp41 is not tolerated for Env function.

Several Env defects have been described in the literature as "dominant negative", based on their potent functional suppressive activities when coexpressed with wild type Env. Mutants in the gp120/gp41 cleavage site, which alone are completely inactive for fusion and infectivity, are reported to have strong dominant negative activities when coexpressed with wild type Env [49,50]. In our previous studies complementation was not detected with mixed trimers in which Unc was one of the defective variants [25]; thus dominant suppression appears to be the major functional activity for uncleaved Env. However we show here that this is not the case for all reported dominant negative mutations. Despite the strong inhibitory activities in fusion and infectivity assays reported for the FP2 mutation when coexpressed with wild type [45], we found that the same mutant is still able to complement fusion activity (albeit at relatively low levels) in mixed trimers with various non-functional Envs (Fig. 4). In fact the relative complementation efficiencies of the FP mutants (FP26 > FP9 > FP2) (Fig. 4) inversely correlated with their previously described inhibitory effects when coexpressed with wild type Env (FP2 > FP9 > FP26) [45]. Another example involves the TM domain, for which it has been reported that substitution of this HIV-1 Env region with its counterpart from the influenza virus hemagglutinin glycoprotein results in potent dominant inhibition [51]. The present studies indicate that this suppressive effect is not due simply to the absence of the native functional HIV-1 TM region, since we observed complementation with fusion-defective constructs in which the normal membrane-spanning domain was replaced by a GPI anchor, or was completely deleted (Fig. 5A). Thus the complementation analyses help distinguish between a fusion-defective Env variant that exerts a strictly dominant suppressive activity, vs. others that, though defective, can permit a low level of functionality as revealed by the ability of their active determinants to complement when coexpressed with a variant defective in another function.

Complementation in cell fusion assays was observed not only with the previously described robust vaccinia-based expression and reporter system (Figs. 2, 3, 4, 5, 6), but also with Env expression from an HIV-1 infectious molecular clone using the MAGI reporter cell line as targets (Table 1). However there were marked variations in complementation efficiencies depending on the assay system employed. Thus, for complementation between LAI-BS and LAI-FP26, the activities in the vaccinia cell fusion system ranged from about ~30–50% relative to wild type WT, consistent with our previous findings [25]; in contrast, the relative activities were much lower with the infectious HIV/MAGI system, i.e. only ~6% in the cell fusion assay and below detection in the virus entry assay (Table 1). We believe these differences mainly reflect variations in the robustness of the functional Env-receptor interactions and the associated reporter gene activation readouts in the different assays, rather than fundamental mechanistic distinctions. Numerous variables can influence the efficiency of Env-receptor interactions leading to fusion/entry, including surface densities of the participating molecules, gp120 affinities for CD4 and coreceptor, varying receptor conformations and molecular associations, the biochemical environments of both effector and target membranes (lipid composition, facilitating or interfering accessory factors), etc. [52,53]. Similarly for the reporter gene readouts, multiple parameters can influence detectability (signal sensitivity, signal/background ratios, etc.), and different factors might be limiting for the measured readout in alternate assay systems. Thus, while a particular assay might be quantitative in terms of yielding numerically reproducible values, such data are not necessarily proportional to the inherent functional activities of the particular Env-receptor interactions involved. Therefore, some assays might reveal weak activities not detected by others, but might overestimate their relative efficiencies. Further complicating the quantitative interpretation is the fact that unlike wild type Env, for which all trimers are potentially active, the complementation activities result only from mixed trimers which presumably represent a subset (theoretically 75%) of the total; moreover upon cotransfection of two nonfunctional Env variants (A & B), the relative functionalities of the two possible mixed trimers (AAB and ABB) might be very different. Thus the reported absence of Env complementation in assays of both reporter virus entry and cell fusion [54] could reflect the absence of functional interactions between the particular mutant Env constructs examined (different from those tested in this report), or limited sensitivities in the assays used. Another point worth noting is that our approach to studying cooperativity within the Env trimer involved complementation analyses between Env mutants that were inactive when expressed alone; thus functional activity was detected despite the presence of a fusion-impairing determinant in every protomer of the mixed trimer. It seems reasonable to propose that the contributions of subunit cooperativity to Env function might be greater with wild type native Env molecules, in which all subunits are fully functional.

We propose that our inability to detect complementation in the virus entry assay despite its clear measurement in the parallel cell fusion assay does not necessarily imply fundamental differences in the corresponding membrane fusion mechanisms. Several inter-related factors presumably contribute to the inability to detect complementation in the virus entry assay. For one, the density of trimeric spikes on HIV-1 virions recently observed by cryo-electron microscopy [55,56] is quite low (<15 trimers per virion, range ~1–3 dozen). A second point concerns uncertainties in the trimer stoichiometry required for Env-mediated virion entry, as indicated by differences in recent effort to fit experimental data to mathematical models. Thus from analyses of pseudotype assays with mixed trimers, it has been concluded in one report that HIV-1 virion-cell fusion requires only a single trimer [57]; by contrast, fitting the same experimental data using alternative models with different underlying assumptions led to conclusions of multi-trimer requirements: ~5, with a wide range of uncertainty in one analysis [58], and ~8 with a range of 2 – 19 in another [59]. According to these multi-trimer mechanisms, an infectious HIV-1 particle does not display a significant excess of functional fusion units. For the complementation analyses described herein where each Env protomer contains a functional defect, it is likely that the complementing trimers are less active than the fully wild type counterparts; moreover as noted above, only a subset of possible trimer forms are likely to be active. Thus in the virus entry assay with complementing Envs, there may be an insufficient number of functional fusion units on most virions, resulting in a major reducton in the fraction of virions with a functional fusion unit. Thus the absence of detectable complementing activity in the virus entry assay need not imply that virus-cell fuson proceeds by a different mechanism than cell-cell fusion. However, we acknowledge that the potential for mechanistic differences is not formally excluded, as emphasized by a recent report arguing that HIV virion entry proceeds by endocytosis and dynamin-dependent fusion out of the endosomes, with direct plasma membrane fusion failing to promote content delivery [60].

Given the experimental complexities, assessing the biological significance of subunit cooperativity for HIV entry is a challenging problem. It is well known that there are functional constraints on subunits within the trimer compared to their monomeric counterparts. A particularly striking example is the interaction of soluble CD4 (sCD4) with gp120; the comparably high affinity of sCD4 for soluble monomeric gp120 from primary and T cell line-adapted HIV-1 isolates stands in marked contrast to the relatively weak binding and neutralization activities of sCD4 for native trimeric Env on the former compared to the latter [61]. Cooperative subunit interactions, whereby binding of gp120 to CD4 on one protomer in the trimer initiates fusion-related conformational changes in the other protomers, might thus enhance Env fusogenic activity, particularly toward target cells containing low densities of CD4 and coreceptor. Another consideration involves the extensively studied phenomenon of epitope masking within the HIV-1 Env trimer [62]. For example, some highly conserved epitopes are freely accessible on monomeric gp120 but are masked in the trimer prior to CD4 binding; cooperative subunit interactions may facilitate exposure of such epitopes on subunits within the trimer that have not yet engaged CD4. With questions such as these in mind, the combination of detailed functional and structural studies will potentially delineate the molecular basis for subunit cooperativity within the native HIV-1 Env trimer, and help define its biological significance.

## Conclusion

The data presented herein demonstrate that every subunit within the Env trimer need not be competent for all critical activities. Cooperatvie cross-talk occurs between subunits, thereby enabling adjacent protomers to complement different functional defects. The diversity of defects that can be complemented illustrates the general nature of cooperative subunit interactions within the HIV-1 trimer. Cooperativity may have important implications for Env function and sensitivity to neutralization.

## Abbreviations

HIV: human immunodeficiency virus; Env: envelope glycoprotein; V3: third variable loop of gp120; FP: fusion peptide; HR: heptad repeat; BS: CD4 binding site; TRR: tryptophan-rich region: TM: transmembrane.

## Competing interests

The authors declare that they have no competing interests.

## Authors' contributions

KS designed and performed experiments, and contributed to data analysis and interpretation, and to writing of the manuscript. EB contributed to data analysis and interpretation, and to the writing of the manuscript.
